# Factors contributing to socio-economic inequality in utilization of caesarean section delivery among women in Indonesia: Evidence from Demographic and Health Survey

**DOI:** 10.1371/journal.pone.0291485

**Published:** 2023-09-13

**Authors:** Pradeep Kumar, Shobhit Srivastava, Pratishtha Chaudhary, T. Muhammad

**Affiliations:** International Institute for Population Sciences, Mumbai, Maharashtra, India; Werabe University, ETHIOPIA

## Abstract

**Background:**

Most of the existing literature in developing countries focused on either the rising trend of CS or its determinants. There is a paucity of population-based studies on existing socioeconomic inequalities in availing CS services by women in Indonesia. This study aimed to assess the factors associated with caesarian section (CS) delivery and explore the various factors contributing to inequalities in CS delivery rates in Indonesia.

**Methods:**

The study utilized nationally representative cross-sectional data from the Indonesia Demographic and Health Survey (IDHS), 2017. We conducted multivariable logistic regression to find the factors associated with CS delivery. Concentration index and Wagstaff’s decomposition analysis were used to examine the socioeconomic inequalities in CS delivery among women and associated factors.

**Results:**

About 17% of women in Indonesia delivered babies through CS. A concentration index of 0.31 in CS delivery rate showed a higher CS delivery rate among women belonging to rich households. About 44.7% of socioeconomic status inequality in CS delivery was explained by educational status among women who went for CS delivery. Women’s place of residence explained 30.1% of socioeconomic inequality, and women’s age at first birth explained about 11.9% and reporting ANC visits explained 8.4% of the observed inequality. Highest socioeconomic inequality was witnessed in central Sulawesi (0.529), followed by Maluku (0.488) and West Kalimantan (0.457), whereas the lowest was recorded in Yogyakarta (0.021) followed by north Sulawesi (0.047) and east Kalimantan (0.171). Education (44.7%) followed by rural-urban place of residence (30.1%) and age of first birth (11.9%) contributed most to explain the gap in CS delivery among rich and poor women.

**Conclusion:**

The study highlighted the higher CS delivery rates among women from higher socioeconomic groups and thus, it is important to frame policies after identifying the population subgroups with potential underuse or overuse of CS method of delivery.

## Introduction

Caesarean section (CS) is a surgical process generally performed when certain complications arise during pregnancy or when normal delivery can put the mother or the baby at risk. However, CS delivery is significantly associated with maternal and perinatal morbidity and also affect future pregnancies [1–3]. The World Health Organization (WHO) recommended the acceptable range of CS rate for a country to be 10–15% at the population level [4]. Despite immediate risks and long term effects, there is a rapid increase in CS rates in the last two to three decades. A study of 150 countries showed that the developed regions such as America and Europe has the highest CS rates, followed by Asia, Africa and other developing countries [2]. A hospital study of four Southeast Asian countries showed, on average, 27 per cent of women had their delivery through CS [1]. Similarly, in Thailand, CS rates at national level has increased from 11.3% in 1992 to 23.6% in 2011 [5]; and at facility level, a study of 24 government hospitals reported 31.4% of CS rates in Khon Kaen province of Thailand [6].

The CS deliveries are requisite in certain medical conditions like excessive bleeding, long period of labour, breech presentations, multiple births, etc. [7]. However, there is a combination of social, cultural and institutional factors that drive the CS rates extremely high [8]. Maternal age, birth order, previous mode of delivery, size of the baby are a few common factors that affect the CS rates [7, 9]. Several studies also showed that urban women with higher education level and higher socio-economic status are more exposed to CS delivery [10, 11]. Previous studies in Indonesia reported that health insurance plays an important role in accessing maternal health care and reducing socioeconomic inequality in access to maternal health services in the country [12, 13]. And education, wealth status, birth parity and awareness on pregnancy complications were found to be major factors determining the ownership of health insurance among women in Indonesia [14, 15]. Health insurance coverage in Indonesia was found to increase the maternal healthcare utilization and chances of deliveries by skilled birth attendants [16].

Existing literature suggests that socioeconomic inequalities in CS rates persist in both, between and within the countries, but there is only very few evidence to show the inequalities in the use of CS among women of different socioeconomic groups. A global study showed that absolute inequality substantially persists in 19 out of 72 countries, and CS rates were highest in the richest women [17]. A study in Indonesia estimated the trends and inequalities in CS rates and found inequalities exist in CS rates across the type of facilities, places of residence, regions, wealth quintile and maternal education. The CS rates are higher in Western Indonesia, the most developed region in the country, comparing to Eastern Indonesia, the least developed region in the country [18]. Another similar study that analysed six rounds of Indonesian DHS data found that richer women were more likely to give birth by CS than their poorest counterparts [19]. In Ghana, the underutilization of CS services among poor women and overutilization among richer women were observed [20]. Similarly, a study in Bangladesh showed there is a rise in CS utilization by women of higher socio-economic status group [21] and very less in poor rural women [22].

Most of the existing literature in developing countries focused on either the rising trend of CS delivery or its determinants. A ten percent increase in CS delivery between the years 2007 and 2017 was observed among Indonesian women and higher socioeconomic status and more antenatal care (ANC) visits were positively associated with CS delivery [23]. However, there is a paucity of population-based studies on factors that contribute to the existing socioeconomic inequalities in availing CS services by women in Indonesia. Therefore, this study aimed to assess the socioeconomic inequality in rates of CS delivery and explore the various factors contributing to such inequalities in CS rates among women in Indonesia. The study hypothesized that there were differentials in CS rates by socioeconomic status of women in Indonesia.

## Materials and methods

### Data

The study utilized nationally representative cross-sectional data from the Indonesia Demographic and Health Survey (IDHS) conducted in 2017. IDHS provided information on demographic and health indicators and carried out by the National Population and Family Planning Board (BKKBN), Statistics Indonesia (BPS), and the Ministry of Health (Kemenkes) [24]. The 2017 IDHS used a two-stage stratified sampling design. In the first stage, census blocks were selected with systematic sampling proportional to size using the sampling frame from the 2010 population census. Further, census blocks were stratified by urban and rural areas and ordered by wealth quintile. In the second stage, 25 ordinary households (HH) were selected with systematic sampling from updated HH listing from each selected census block. Details of the survey design and data collection procedure have been published elsewhere [24]. The data are freely available in public domain and survey agencies that conducted the field survey for the data collection have collected a prior consent from the respondent. Local ethics committee of Statistics Indonesia ruled that no formal ethics approval was required to carry out research from this data source.

Among total 47,963 households, 49,627 eligible women and 10,009 eligible men were interviewed with 99.5%, 97.8%, and 95.9% response rate respectively. The sample size of this study was 17,848 women who had live births in the five years preceding the survey.

### Outcome variable

The outcome variable of this study was CS delivery, which is a surgical procedure in which the infant is born through an incision in the mother’s abdomen and womb. The question was asked to the women: ‘was (name) delivered by CS?’ that is, did they cut your belly open to take the baby out? The responses were recorded as either ‘Yes’ or ‘No.’ The question on CS delivery was asked only to those women who delivered in a health facility.

### Predictor variables

The explanatory variables used in this study were age (≤24 years, 25–29 years, 30–34 years and ≥35 years), education (no education, primary, secondary, and higher), birth order (1^st^, 2^nd^, 3^rd^ and 4 or more), type of facility (public and private), age at first birth (<18, 18–20, 21–24 and ≥25 years), number of antenatal care visits (no visit, 1–4 visits and 5 or more visits). The question used to assess the number of ANC visits was “How many times did you receive antenatal care during this pregnancy?”. We also include the size of the child at birth (very large and larger than average were merged as 1 ’’Larger than average", “average” and smaller than average and very small were merged as 3 "Smaller than average"). The question used to assess the size of the child at birth was “When (NAME) was born, was (NAME) very large, larger than average, average, smaller than average, or very small?” Type of birth (single and multiple), place of residence (urban and rural), regions (Sumatera, Java, Bali and Nusa, Tenggar, Kalimantan, Sulawesi and Maluku and Papua) and wealth quintiles (poorest, poorer, middle, richer, and richest) were also included as covariates in this study.

### Equity stratifier

In IDHS, the wealth quintile was the key variable to measure the economic status of the household. The survey constructed the wealth quintile based on household characteristics, household assets, and possession of durable goods using principal component analysis (PCA). A national-level wealth quintile was obtained by assigning the household score to each household member, ranking each person in the population by his/her own score. The study used this score for decomposition analysis and the calculation of the concentration index (CI); the study divided the ranking into five equal categories, each comprising a 20% population.

#### Statistical analysis

We performed descriptive analysis, and binary logistic regression analysis [24] was used to find the association of CS rates with background characteristics. Additionally, the variance inflation factor (VIF) [25–27] was measured to assess the multicollinearity, and it was revealed that there was no evidence of multicollinearity in the variables used. Svyset command was used in STATA 14 to account for complex survey design. Further, individual weights were used to make the estimates nationally representative. STATA 14 [28] was used to analyse the dataset.

#### Concentration index

Concentration index represents the magnitude of inequality by measuring the area between the concentration curve and line of equality and calculated as twice the weighted covariance between the outcome and fractional rank in the wealth distribution divided by the variable mean [29, 30].

The concentration index can be written as follows:

$$C=\frac{2}{\mu }cov(y_{i},R_{i})$$

Where C is the concentration index; *y*_*i*_ is the outcome variable index; ***R*** is the fractional rank of individual ***i*** in the distribution of socio-economic position; ***μ*** is the mean of the outcome variable of the sample, and ***cov*** denotes the covariance [31]. The index value lies between -1 to +1.

If the curve lies above the line of equality, the concentration index takes a negative value, indicating a disproportionate concentration of CS delivery taking place among the poor (pro-rich). Conversely, if the curve lies below the line of equality, the concentration index takes a positive value, indicating a disproportional concentration of CS delivery taking place among the rich (pro-poor). In the absence of such a socio-economic-related inequality, the concentration index is zero. Further, the study decomposes the concentration index to understand the relative contribution of various factors to the gap in CS delivery rates among women from the poor and rich households. To do this, the study used the regression-based decomposition technique [30, 32, 33], which was proposed by Wagstaff et al. [34]. In this model, CS delivery is considered the outcome variable. The decomposition will be based on the logistic regression relationships between the outcome variable *y*_*i*_, the intercept α, the relative contribution of *x*_*ki*_ and the residual error *ε*_*i*_

$$y_{i}=\alpha +\sum \beta_{k}x_{ki}+\varepsilon_{i}$$

Where *ε*_*i*_ is an error term, and given the relationship between *y*_*i*_ and *x*_*ki*_, the CI for y (C) can be rewritten as:

$$C=\sum \left(\frac{\beta_{k}{\bar{x}}_{k}}{\mu }\right)C_{k}+\frac{GC\varepsilon }{\mu }/\mu$$

Where *μ* is the mean of *y*_*i*_, ${\bar{x}}_{k}$, is the mean of *x*_*k*_, *β*_*k*_ is the coefficient from a linear regression of outcome variable, *C*_*k*_ is the concentration index for *x*_*k*_ (defined analogously to C, and GC_ɛ_ is the generalized concentration index for the error term (*ε*_*i*_).

Here C is the outcome of two components: First, the determinants or ‘explained’ factors. The explained factors indicate that the proportion of inequalities in the outcome (Cesarean delivery) variable is explained by the selected explanatory factors, i.e., x_k_. Second, a residual or ‘unexplained’ factor $\left(\frac{GC\varepsilon }{\mu }/\mu \right)$, indicating the inequality in health variable that cannot be explained by selected explanatory factors across various socio-economic groups [35].

## Results

Table 1 depicts the percentage distribution of background characteristics of surveyed women in Indonesia. Almost 17% of women had CS deliveries, which is higher than recommended by WHO [4]. About one-fifth of women in the study belonged to the age group 24 years or less. Only 1.2% of women had no education, whereas 15.5% of women had a higher education. Almost 13% of women were children of order four or more. About every second women who delivered birth in a private facility went for CS delivery. One-tenth of women were aged below 18 years at their first birth. Only 3% of women did not report ANC visits, whereas more than 86% of women reported five or more visits. Almost 31% of women whose new-born children were having a size larger than average, and 14% of women whose children had lesser size. Only 1.3% of women delivered multiple babies. Half of the women belonged to rural areas. Nearly 53% of women belonged to Java, followed by Sumatera (23%) and Sulawesi (7.2%).

**Table 1:** Weighted percentage distribution of women by selected background characteristics, Indonesia, 2017.

Covariates	N = 17,019
	N (Percentage)
**CS delivery**	2,896 (17.0)
**Age (in years)**	
≤24	3190 (18.7)
25–29	4367 (25.7)
30–34	4460 (26.2)
≥35	5003 (29.4)
**Education**	
No education	198 (1.2)
Primary	4397 (25.8)
Secondary	9795 (57.6)
Higher	2629 (15.5)
**Birth order**	
1^st^	5963 (35.0)
2^nd^	5731 (33.7)
3^rd^	3086 (18.1)
4^th^	2239 (13.2)
**Type of facility**	
Public	5176 (30.6)
Private	8271 (48.8)
**Age at first birth (in years)**	
<18	1775 (10.4)
18–20	4830 (28.4)
21–24	5898 (34.7)
≥25	4516 (26.5)
Anti-Natal Care#	
No visit	457 (3.0)
1–4 visits	1564 (10.4)
5 or more	12995 (86.5)
**Size at Birth**	
Smaller than average	2291 (13.6)
Average	9437 (55.8)
Larger than average	5185 (30.7)
**Type of birth**	
Single	16793 (98.7)
Multiple	226 (1.3)
**Wealth Index**	
Poorest	3518 (20.7)
Poorer	3422 (20.1)
Middle	3419 (20.1)
Richer	3438 (20.2)
Richest	3222 (18.9)
**Residence**	
Urban	8257 (48.5)
Rural	8762 (51.5)
**Region**	
Sumatera	3966 (23.3)
Java	9077 (53.3)
Bali and Nusa Tenggar	1099 (6.5)
Kalimantan	1066 (6.3)
Sulawesi	1219 (7.2)
Maluku and Papua	593 (3.5)

#The variable information was asked for last birth only

Table 2 presents the prevalence and odds ratio of CS delivery by background characteristics. Women aged 35 and above had significantly higher odds of a delivery by CS than women aged 24 years or less [Odds ratio (OR):2.30, p<0.01]. Odds for CS deliveries were significantly higher among women who were having educational status higher and above than those who were not educated [OR: 3.03, p<0.01]. Interestingly, women who delivered babies at the public facility were 37% significantly more likely to deliver through CS than women who delivered babies at the private facility [OR: 1.37, p<0.01]. Women whose age at first birth was 25 years and more were having a 72% significantly higher odds of CS delivery than women whose age at first birth was less than 18 years [OR:1.72, p<0.01]. Odds for CS was high among women who reported five or more ANC visits than women who reported no ANC visits [OR: 2.76, p<0.01]. Women who gave birth to multiple babies had higher odds of CS delivery than women who gave birth to a single child [OR: 3.19, p<0.01]. Women from urban areas had a 38% significantly higher odds of CS delivery than women from rural areas [OR: 1.38, p<0.01]. Women from the Sumatera region had 57% higher odds of CS delivery than women from Maluku and Papua region [OR: 1.57, p<0.01].

**Table 2:** Prevalence and odds ratio of CS delivery by background characteristics of women, Indonesia, 2017.

Covariates	CS delivery		AOR (95% CI)
**Age (in years)**		$	
≤24	12.0		Ref
25–29	14.4		1.11(0.93–1.32)
30–34	18.7		1.48***(1.21–1.81)
≥35	21.3		2.13***(1.70–2.67)
**Education**		$	
No education	2.6		Ref
Primary	9.0		1.20(0.58–2.47)
Secondary	16.9		1.45(0.71–30)
Higher	32.5		2.0**(0.96–4.15)
**Birth order**		$	
1^st^	18.9		Ref
2^nd^	17.0		0.70***(0.61–0.80)
3^rd^	17.0		0.63***(0.52–0.75)
4^th^	12.8		0.59***(0.47–0.73)
**Type of facility**		$	
Public	21.4		1.37***(1.24–1.52)
Private	21.7		Ref
**Age at first birth**		$	
<18	8.1		Ref
18–20	11.4		1.14(0.92–1.41)
21–24	16.0		1.23**(0.99–1.51)
≥25	28.3		1.72***(1.36–2.18)
**Anti-Natal Care visits#**		$	
No visit	2.1		Ref
1–4	9.8		2.05**(1.13–3.73)
5 or more	19.0		2.49***(1.39–4.47)
**Size at Birth**			
Smaller than average	18.7		1.44***(1.26–1.65)
Average	16.2		Ref
Larger than average	18.1		1.16***(1.05–1.29)
**Type of birth**		$	
Single	16.8		Ref
Multiple	38.4		3.05***(1.97–4.72)
**Wealth Index**		$	
Poorest	6.12		Ref
Poorer	11.37		1.30***(1.09–1.56)
Middle	15.25		1.53***(1.28–1.83)
Richer	21.95		1.97***(1.64–2.37)
Richest	31.98		2.64***(2.18–3.21)
**Residence**		$	
Urban	22.7		1.13**(1.02–1.26)
Rural	11.9		Ref
**Region**		$	
Sumatera	19.0		1.41***(1.13–1.76)
Java	18.0		0.83(0.66–1.04)
Bali and Nusa Tenggar	15.5		0.74**(0.57–0.96)
Kalimantan	13.1		0.93(0.72–1.21)
Sulawesi	13.6		0.90(0.71–1.14)
Maluku and Papua	8.3		Ref

**Ref**: reference category; $: p<0.001 chi-square test; ***p<0.0001; **p<0.05; AOR: Adjusted Odds Ratio; CI: confidence interval; #The variable information was asked for last birth only

**Fig 1:**
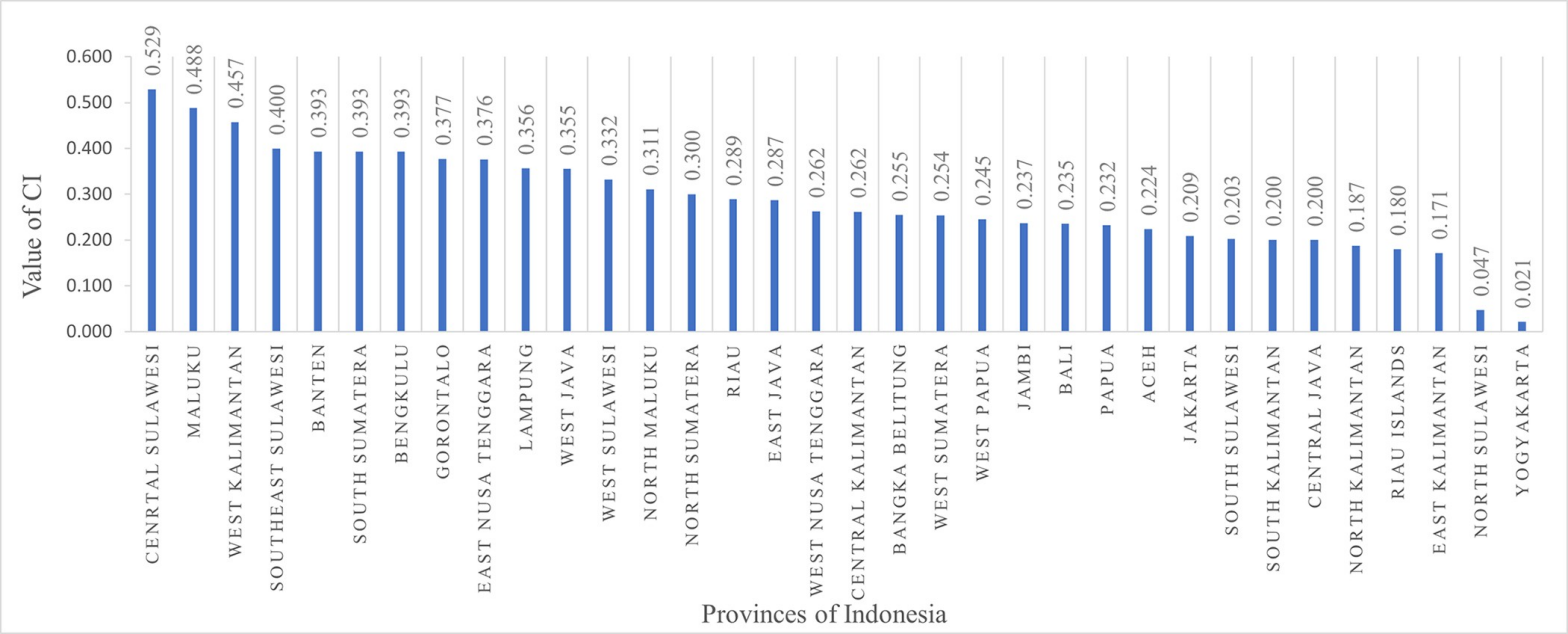
Concentration index for CS delivery among women in provinces of Indonesia.

Fig 1 depicts the value of the concentration index for each state of Indonesia. The highest socioeconomic inequality in CS delivery rates was witnessed in central Sulawesi (0.529), followed by Maluku (0.488) and West Kalimantan (0.457), whereas the lowest was recorded in Yogyakarta (0.021) followed by north Sulawesi (0.047) and east Kalimantan (0.171).

**Fig 2:**
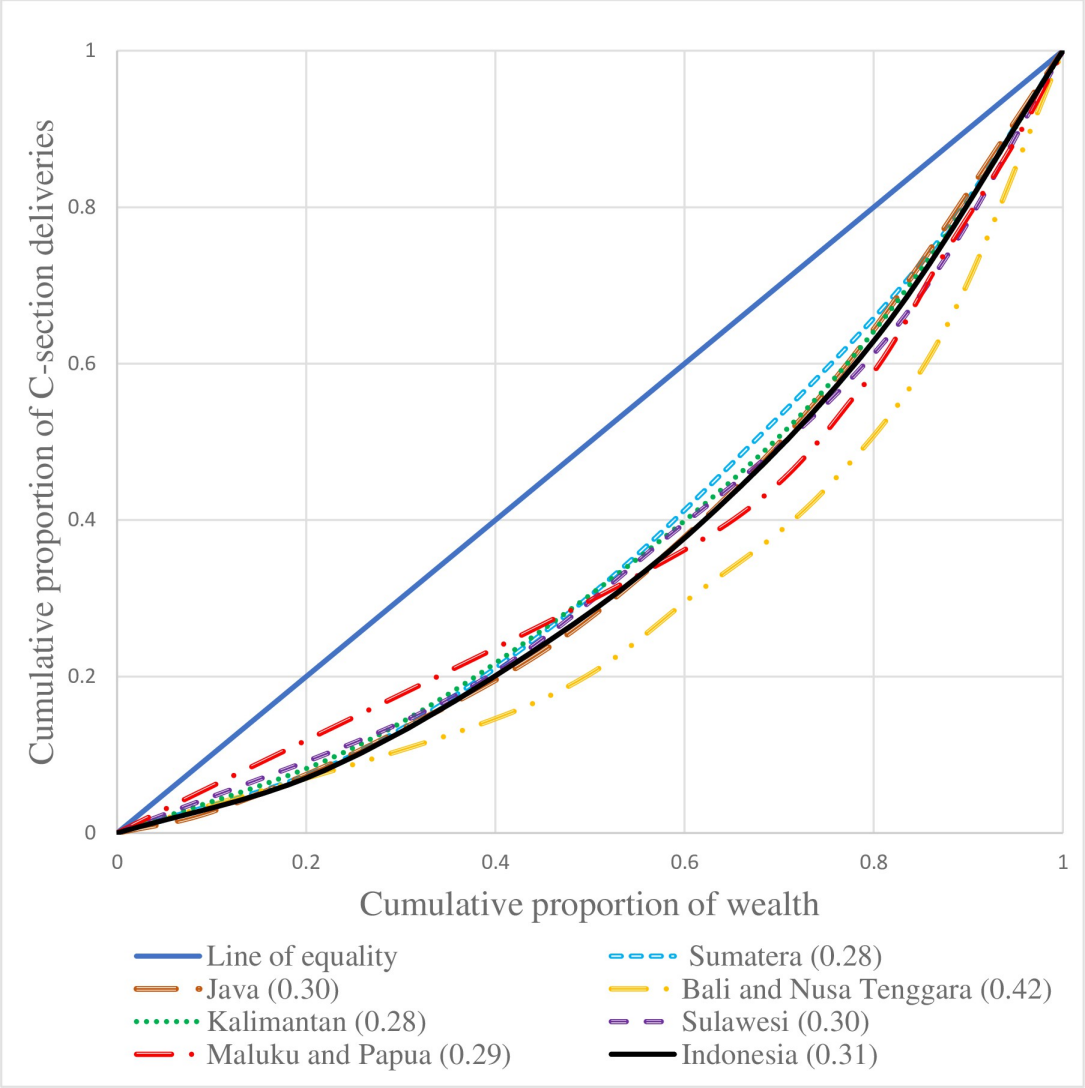
Concentration curve for CS deliveries in Indonesia and its regions.

Fig 2 depicts the concentration curve for six regions of Indonesia and Indonesia as a whole for CS deliveries. If the curve is formed below the line of equality, then the inequality is concentrated towards the rich and vice-a-versa. Additionally, the more the area between the line of equality and curve represents the higher inequality. The regions of Bali and Nusa Tenggara (0.42) were having the highest socioeconomic inequality in CS delivery rates, followed by Java (0.30) and Sulawesi (0.30); and on average, Indonesia was having an inequality of 0.31 showing a higher CS delivery rate among women belonging to the rich households.

Table 3 depicts the contribution of various factors to the observed socioeconomic inequality in CS delivery based on the decomposition of concentration index. The positive scores of CI denote that the women with the characteristics in question were highly represented among the rich category and vice-versa. The value of absolute contribution indicates the extent of inequality contributed by the explanatory variable. About 44.7% of socioeconomic status inequality in CS delivery was explained by educational status among women who went for CS delivery. Women’s place of residence explained the 30.1% of socioeconomic inequality, and women’s age at first birth explained about 11.9% and reporting ANC visits explained 8.4% of the observed inequality, and the regions of Indonesia explained 2% of socioeconomic inequality in CS delivery among women.

**Table 3:** Contribution of determinants based on the decomposition of concentration index analysis for CS delivery among women, Indonesia-2017.

Covariates	Elasticity	Concentration Index	Absolute contribution	Percentage contribution	
**Age (in years)**					6.8
≤24					
25–29	0.002	0.008	0.000	0.05	
30–34	0.014	0.056	0.001	2.43	
≥35	0.033	0.042	0.001	4.29	
**Education**					44.7
No education					
Primary	0.003	-0.353	-0.001	-3.28	
Secondary	0.039	0.038	0.001	4.59	
Higher	0.028	0.501	0.014	43.43	
**Birth order**					3.2
1^st^					
2^nd^	-0.015	0.033	0.000	-1.53	
3^rd^	-0.011	0.034	0.000	-1.16	
4^th^	-0.01	-0.19	0.002	5.88	
**Type of facility**					-5.4
Public	0.021	-0.083	-0.002	-5.40	
Private					
**Age at first birth (in years)**					11.9
<18					
18–20	0.001	-0.131	0.000	-0.41	
21–24	0.004	0.031	0.000	0.38	
≥25	0.019	0.202	0.004	11.88	
Antenatal Care#					8.4
No visit					
1–4 visits	0.004	-0.272	-0.001	-3.37	
5 or more	0.076	0.05	0.004	11.76	
**Size at Birth**					-1.7
Smaller than average	0.005	-0.119	-0.001	-1.84	
Average					
Larger than average	0.006	0.01	0.000	0.19	
**Type of birth**					-0.0
Single					
Multiple	0.003	-0.002	0.000	-0.02	
**Residence**					30.1
Urban	-0.038	-0.256	0.010	30.11	
Rural					
**Region**					2.0
Sumatera					
Java	0.018	-0.093	-0.002	-5.18	
Bali and Nusa Tenggar	0.020	0.152	0.003	9.41	
Kalimantan	0.001	-0.279	0.000	-0.86	
Sulawesi	0.002	-0.108	0.000	-0.67	
Maluku and Papua	0.001	-0.223	0.000	-0.69	
**Explained CI**			0.032	100	
**Actual CI**			0.31		
**Residual**			0.28		

CI: Confidence Index; **ANC:** Antenatal care

#The variable information was asked for last birth only

## Discussion

CS delivery is a surgical procedure that can reduce maternal and infant mortality as well as several pregnancy-related complications. It is mostly carried out based on medical indication, and it can be performed in emergency conditions or in planned cases. The current study found a higher rate of CS delivery with a proportion of 17% of women in Indonesia having a CS delivery. There could be numerous reasons for this higher CS rate, such as social correlates and fear of pain during labour and childbirth [36, 37], previous experience and interactions with health care professionals [38], doctors’ advice [9, 39], changes in lifestyle leading to obesity [40], increase in the mother’s age at first birth [41], the role of private facilities to make more money [42–44] from CS as it requires less time and a smaller health workforce etc.

A higher rate of CS deliveries was found among women with higher socio-economic profile in the study in comparison to women from lower socio-economic groups. The results are parallel to other studies which stated that overuse of CS methods was witnessed among rich and highly educated women [20, 45, 46]. The reason for women from higher socio-economic profile to go for CS may be due to the cost associated with it [47]. The CS-related cost is the major correlate preventing poor women from accessing CS at health facilities [47–49]. The association of CS delivery and women’s birth order is a well-established fact. It was found in the present study that women with higher birth order have less likelihood of undergoing CS delivery. The results are consistent with previous research in Pakistan that showed that when women first go for childbirth; they have a higher likelihood of having a CS [50]. The plausible reason is that women who go for first childbirth may have a fear of labour and opt of CS [50, 51].

Another interesting finding of this study is that the odds of CS deliveries were high in public facilities compared to private facilities in Indonesia. Earlier research observed contradictory findings compared to the present study reporting higher rates of CS deliveries in private facilities [52]. A previous study using seven rounds of IDHS found that CS deliveries were higher in public facilities except in the recent round of IDHS in the year 2017, when the CS rates were similar across private and public facilities [18]. The increasing trend in higher CS rates in public facilities may be attributed to the health insurance schemes and the increased availability and accessibility of CS services which is a reflection of increased investments in health infrastructure and overall health system development in the country [48]. However, a study from Malaysia found that CS rates are rising significantly in public health facilities [53]. Further research is needed in this domain to explore the reasons for higher CS rates in public health facilities with special focus on low- and middle-income countries.

As we observed in the present study, women who had five or more ANC visits had higher odds of CS delivery. This is in agreement with previous study suggesting that women with more ANC visits had higher chances of having pregnancy-related complications and, therefore, may have higher chances of going for CS delivery [54]. The size of babies at birth is another factor for increased CS among women. It was argued that babies who weigh more were more likely to have labors that eventually end in a CS delivery [55]. A similar result was also found in the previous literature arguing that Cephalo-pelvic disproportion commonly associated with fetal macrosomia may explain the positive association of large birth size with higher CS rates [56]. Similarly, advanced maternal age at first birth was one of the contributing factors for CS births [57]. The possible reasons include pre-pregnancy morbidity and higher morbid conditions due to advanced age among women [57]. Further, women who gave birth to multiple babies were at higher risk of delivering through CS. The reason is that with multiple births, there is an associated risk of complications, like perinatal mortality and to overcome these problems, the CS method is suggested by health professionals [58]. Apart from all these factors, elective caesarean, i.e. opting for cesarean by women in the absence of medical indications, is also playing an important role in raising CS rates. Community perception regarding CS as safer and beneficial in the survival of women and newborns is also leading women to go for caesarean by choice [59]. These factors need to be investigated in future research.

Further, in the present study, it was found that the higher rate of CS was concentrated among women who had higher education. Moreover, women from an urban place of residence used to utilize more CS method. The probable reason was that in Indonesia, people living in urban areas accessed health facilities seven times more than their rural counterparts [60]. Additionally, urban areas in Indonesia are better equipped with medical facilities than rural areas and hence may be probably the reason for such inequality [60]. Decomposition analysis also showed that education and place of residence are the major drivers of inequality in CS delivery. The results were consistent with previous literature stating the similar results [21]. Additionally, women who go for five or more ANC visits and whose age at first birth was 25 years and more were having a higher concentration of CS deliveries. The finding is similar as previously discussed, that rich women have more access to ANC than poor women. Moreover, women with pregnancy complications could be one of the most important reasons for the preference of delivery though CS [61]. As age increases, the risk of pregnancy complications also increases, which leads to the higher chances of opting for CS by advanced age women [57] as it was found that the increased age at CS delivery was more likely to be concentrated among women from higher socio-economic groups and vice versa [62].

The current findings showed that women from the Sumatra region had the highest prevalence of CS delivery than any other regions in Indonesia. The reason for the high prevalence in this region is unknown but can be attributable to its better socio-economic profile [63]. Despite drastic increment in CS delivery rates, access to obstetric care is a major concern in Indonesia and is still a big challenge for many of the developing countries [17, 20–22]. Indonesia falls in the category of a low and middle-income country struggling to provide basic maternal health care equally to all women. Though several maternal and child health (MCH) indicators show overall improvement, it is far away from achieving the Millennium Development Goal-5 (MDG-5) of maternal mortality ratio (MMR) [64]. Along with the institutional deliveries, Indonesia’s health system also focused on providing skilled birth care in communities and homes as well [65]. However, the quality of emergency obstetric care is not good enough [64], and inequity is its key constraint [19], and recent studies reported a higher proportion of mothers from poor socioeconomic group delivering in in home or other setting [66]. Therefore, socioeconomic differences in facility-based delivery should be the focus of future research and health-decision and policy making in the country.

Major limitation of the current study is its cross-sectional design which abolishes from establishing the causal relationships. Further, the IDHS data provide the information about whether the delivery was normal or caesarean, and if it was caesarean, when and why the decision of caesarean was taken is not available, which can give more ideas about emergency caesarean over elective caesarean delivery. Hence, from available information, it’s quite difficult to list out the correlates contributing to increasing CS rates. Moreover, since many of them are self-reported, the obstetric correlates reported in DHS data will not be as accurate as hospital-based data–as women may not be fully informed on why they received CS (especially in low and middle-income countries (LMICs)) and subsequently may under/over report their risks. However, this study is one of the few such studies in Indonesia, which talks about increasing CS rates and inequalities in availing CS services, which may prompt further in-depth research in this area.

## Conclusion

Socioeconomic inequalities in CS delivery rates observed in the study show that considerable differences in CS utilization persist in Indonesia and within the country (provinces). Bali and Nusa Tenggara region reported the highest inequality, showing a higher CS delivery rate among women belonging to the rich households. Further, the utilization of caesarean section is mostly concentrated among women with high SES. The study highlighted the higher rates of CS delivery among women from higher socioeconomic background and thus, it is important to frame policies after identifying the population subgroups with potential underuse or overuse. The government needs to ensure that this life-saving procedure should be utilized when it is necessary irrespective of women’s socioeconomic status, and spread knowledge to women and health providers about the risks of adverse outcomes of unnecessary CS delivery.

## Abbreviations

CS: Caesarean section
OR: Odds Ratio
CI: Confidence interval
ANC: Ante-natal care
IDHS: Indonesia Demographic and Health Survey
HH: Household
WHO: World Health Organization
MCH: Maternal and Child Health
MDG: Millennium Development Goal
MMR: Maternal Mortality Ratio
DHS: Demographic and Health Survey
LMICs: Low and Middle-Income Countries
